# What Do Cognitive Networks Do? Simulations of Spoken Word Recognition Using the Cognitive Network Science Approach

**DOI:** 10.3390/brainsci11121628

**Published:** 2021-12-10

**Authors:** Michael S. Vitevitch, Gavin J. D. Mullin

**Affiliations:** Department of Psychology, University of Kansas, Lawrence, KS 66045, USA; gavin.mullin@ku.edu

**Keywords:** phonology, network science, one-phoneme metric, phonological neighbors, spoken word recognition, computer simulation, TRACE, cognitive network

## Abstract

Cognitive network science is an emerging approach that uses the mathematical tools of network science to map the relationships among representations stored in memory to examine how that structure might influence processing. In the present study, we used computer simulations to compare the ability of a well-known model of spoken word recognition, TRACE, to the ability of a cognitive network model with a spreading activation-like process to account for the findings from several previously published behavioral studies of language processing. In all four simulations, the TRACE model failed to retrieve a sufficient number of words to assess if it could replicate the behavioral findings. The cognitive network model successfully replicated the behavioral findings in Simulations 1 and 2. However, in Simulation 3a, the cognitive network did not replicate the behavioral findings, perhaps because an additional mechanism was not implemented in the model. However, in Simulation 3b, when the decay parameter in *spreadr* was manipulated to model this mechanism the cognitive network model successfully replicated the behavioral findings. The results suggest that models of cognition need to take into account the multi-scale structure that exists among representations in memory, and how that structure can influence processing.

## 1. Introduction

Various metaphors have been used to increase our understanding of the mind, with the computer perhaps being the most well-known and fundamental metaphor in Cognitive Psychology [1]. Another metaphor that has been used repeatedly by Cognitive Psychologists to examine representations and processing of various kinds is a “network” of some sort. An early use of the network metaphor in Cognitive Psychology is exemplified in the spreading activation theory of semantic memory proposed by [2]. They suggested that information stored in semantic memory—such as perceptual features (e.g., colors) and common nouns (e.g., fire engine)—could be represented as nodes, and relationships among nodes could be represented by labeled connections between nodes (e.g., “IS-A” and “HAS” links to indicate that a fire engine IS-A type of vehicle and HAS the color red). The spreading of activation across the semantic network proposed by Collins and Loftus has been used to understand numerous memory and language phenomena.

Another use of the network metaphor in Cognitive Psychology is the “artificial neural network” approach exemplified in (localist) connectionist models and in parallel distributed processing (PDP) models. Both types of artificial neural network saw a rise in popularity in the late 1980s and early 1990s [3,4].

In localist connectionist models, nodes represent specific pieces of information, such as a phoneme, a syllable, or a word, and connections link together those pieces of information, often in a hierarchical manner. For example, nodes representing the phonemes /k/, /æ/, and /t/ would be connected to nodes representing words such as *at*, *cat*, *tack*, etc. Those word nodes, in turn, might be connected to another layer of nodes that contain semantic information.

In contrast, in the PDP approach, “…active representations in the mind are thought to correspond to the *patterns of activation* generated over a set of units” [4] (pg. 1038; emphasis added). In this approach, knowledge in memory “…does not exist as a set of dormant data structures in a separate store but is encoded directly in the network architecture, in the values of the connection weights that allow the system to generate useful internal representations and outputs” [4] (pg. 1039). In other words, representations are not symbols stored in a separate memory store (as in the localist connectionist approach), but instead are ephemeral and emerge from the processing that occurs over the many distributed processing units in this type of artificial neural network. The artificial neural network approach was (and remains) a significant driver of research on memory, speech production [5,6], and spoken word recognition [7,8,9].

**Figure 1:**
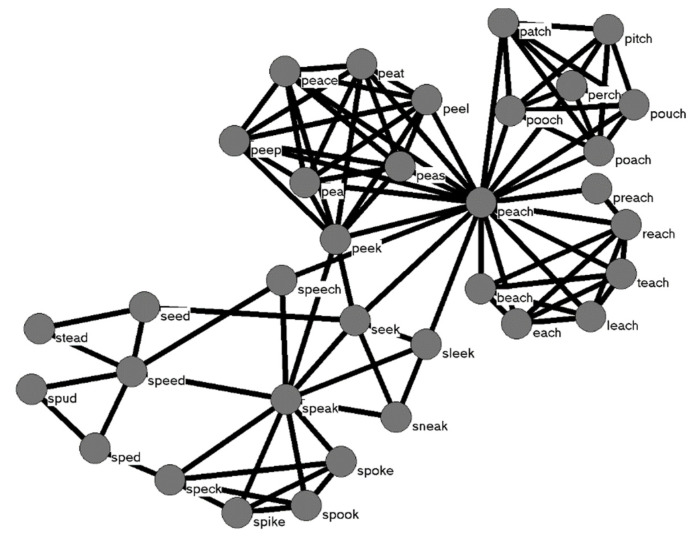
An example of a phonological network where nodes represent words in the mental lexicon, and edges connect words that are phonologically similar to each other (based on the addition, deletion, or substitution of a phoneme in one word to form another word). Phonological similarity can be defined in other ways as well. This network represents the 2-hop neighborhood of the word speech; a 2-hop network contains the neighbors of a word, and the neighbors of the neighbors.

A more recent use of the network metaphor in Psychology can be found in what is becoming known as Cognitive Network Science [10,11]. The Cognitive Network Science approach applies the quantitative tools of network science [12]—used to understand a wide range of complex systems—to address questions about human cognition. In this approach, networks are used to map the relationships that exist among representations stored in memory. In this case, the term network is not referring to an artificial neural network as described above. In the Cognitive Network Science approach, the network consists of *nodes* that represent entities in a system, and *edges* that connect nodes that are related in some way. Cognitive Networks have been used to represent words in the mental lexicon that are semantically related [13], but this approach differs from the earlier semantic network in [2], because the links in cognitive networks are not labeled in the same way as they are in [2] (e.g., “IS-A” and “HAS” links to indicate that a fire engine IS-A type of vehicle and HAS the color red). Rather, the connections between nodes in cognitive networks typically represent a single type of relationship, such as the words being semantic associates of each other [13]. Cognitive networks such as this can also be used to represent other types of information and relationships among words, such as words that are phonologically related, as in Figure 1 [14].

Important to the Cognitive Network Science approach is the fact that the way in which these representations are organized or structured in memory influences how effectively and efficiently processes operate in the system [15,16]. That is, two networks with the same number of nodes and the same number of connections that are just connected in different ways in the two networks will have drastically different outcomes for a simple search algorithm [17]. This central tenet of the Cognitive Network Science approach contrasts with the semantic network of [2] and the artificial neural network approaches, which do not make this assumption, nor measure the structure of their respective types of “networks” in the manner described below.

The structure of a cognitive network can be measured at multiple scales: micro, macro, and meso. The micro-scale refers to measures of individual nodes in the network. Macro-scale measures assess the whole network. At the meso-scale, measures are made of subsets of nodes in the network. Because the structure of a network can influence processing, it is important to measure a cognitive network at all three scales, and to examine how the structure at each scale might influence cognitive processing.

The results of a number of behavioral experiments using conventional psycholinguistic tasks in laboratory settings have shown that certain network structures at various scales of the phonological network influence the production, recognition, and learning of spoken words in English. For example, the experiments in [18] considered a micro-scale measure, the (local) clustering coefficient, and how it influenced spoken word recognition (see also [19,20,21,22]). At the macro-scale, experiments by [23] examined how the location of words in the giant component (i.e., the largest group of connected nodes in a network) or in “lexical islands” (i.e., smaller groups of words that are connected to each other, but not to words in the giant component) of the phonological network influenced spoken word recognition. Finally, at the meso-scale, [24] found that a set of words in key positions, whose removal would disconnect the network, tended to be recognized more quickly than foil words that were similar to the keywords in a variety of lexical characteristics.

Given that the structure of the network influences processing, behavioral studies as well as a computer simulation with an artificial neural network—namely the TRACE model [7]—further demonstrated the importance of considering how nodes in a network are organized [18]. TRACE has been described as “…arguably the most successful model of spoken word recognition (SWR) to date” [25] (pg. 19). Indeed, as of 13-NOV-2021, the paper by [7] was cited over 3650 times (as per Google Scholar).

TRACE is a localist artificial neural network that contains processing units organized into three layers: (1) units representing acoustic–phonetic-like features, (2) units representing phonemes, and (3) units representing words. The units in each layer are excited or inhibited based on how well they match the speech input that is presented to the model. For more details about the TRACE model, we refer the reader to the original work [7], and to the more recent implementation of TRACE, dubbed jTRACE [25], which is used in the simulations reported below.

Twenty-eight monosyllabic words with three phonemes with higher clustering coefficients and 28 monosyllabic words with three phonemes with lower clustering coefficients were selected by [18] from the *initial_lexicon* that was used in the original simulations of TRACE. Using the default parameters, the model ran for 180 time-cycles [18]. At the end of the 180 time-cycles, the difference in the maximum activation levels for words with higher (*mean* = 0.55, *SD* = 0.010) compared to lower clustering coefficient (*mean* = 0.55, *SD* = 0.004), was not statistically significant (*F* (1,54) = 2.012, *p* = 0.16; as reported in [18]).

When the maximum activation levels were reached was also examined [18], and the difference in the number of time-cycles required to reach maximum activation also was not statistically significant (*F* (1,54) = 1.294, *p* = 0.26). As reported in [18], words with high clustering coefficient reached maximum activation on average in the 105th cycle (*SD* = 16.28), and words with a low clustering coefficient reached maximum activation on average in the 99th cycle (*SD* = 17.98). In combination with the behavioral data that they obtained, [18] viewed the inability of TRACE to simulate the results of their behavioral experiments as an indication that the structure of the phonological lexicon is in fact important to consider in models of spoken word recognition. Specifically, as suggested by the Cognitive Network Science approach, the structure of the phonological lexicon influences lexical processing.

Despite the success of the cognitive network approach in accounting for certain aspects of spoken word recognition (and other language-related and memory processes) this approach has been criticized because “…these networks do not ‘do’ anything; they have no function” [26] (pg. 16). One could argue that what cognitive networks “do” is capture in their structure certain regularities and relationships among entities in the world. By adding a simple process such as a random walk or the diffusion of activation across the network, one can examine how the structure of the network at multiple scales might influence cognitive processing.

The three behavioral experiments described above, which demonstrated that human performance in language-related tasks is influenced by structural characteristics at various scales in the phonological network, were simulated in the leading model of spoken word recognition—TRACE [7] (more recently implemented in Java as jTRACE by [25])—and on a cognitive network model based on the phonological network of [14]. If the structure at various scales of the phonological network influences processing, as claimed in the Cognitive Network Science approach, then we expect the phonological network model to qualitatively replicate the results of the three behavioral experiments described above. Further, given that the way in which words are connected to each other (i.e., how the lexicon is structured) is not considered in TRACE, we expect TRACE to fail to qualitatively replicate the results of the three behavioral experiments described above, as it did in [18].

In the Cognitive Network Science approach, edges are used to capture some sort of relationship between nodes resulting in a network that maps the structural organization of information in memory. Processing in these structural models can be modeled by either a random walk [27] or the diffusion of activation—akin to spreading activation—across the network [28]. An *R* package called *spreadr* has recently been created that can diffuse activation across a network provided by the user over a range of timesteps, initial activation levels, etc. [29]. We used *spreadr* in the simulations that follow to diffuse activation across the network from [14], allowing us to examine if a cognitive network can account for the results from the three behavioral experiments that previously examined the influence of the structure of the phonological network at various scales on human language processing.

## 2. Simulation 1: Clustering Coefficient

**Figure 2:**
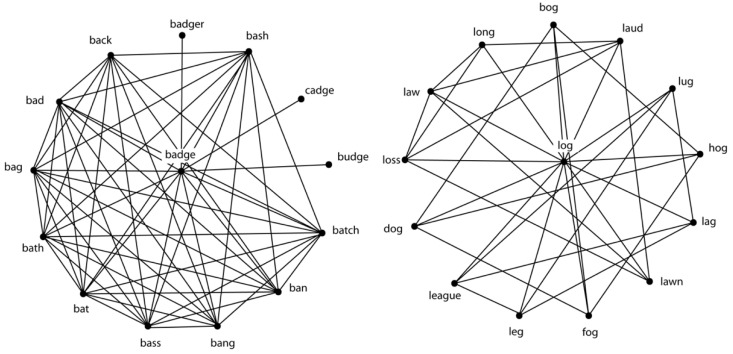
Although both words have the same number of phonological neighbors, the left panel represents a word with a higher clustering coefficient (*badge*), whereas the right panel represents a word with a lower clustering coefficient (*log*). That is, there is a difference in the extent to which the neighbors of each word are also neighbors of each other.

The first study to demonstrate that one of the network structures observed in [14] influenced the performance of humans in a conventional psycholinguistic task was reported in [18]. In that study it was observed that the micro-scale measure known as the (local) clustering coefficient influenced the accurate identification of words presented in noise in a perceptual identification task. Informally and in the context of a phonological network, the local clustering coefficient refers to the extent to which phonological neighbors of a given word are also neighbors of each other (see [18] and others for a more formal definition of clustering coefficient). As seen in Figure 2, the words *badge* and *log* in the middle of the two networks represent the target words, with the same number of phonological neighbors encircling each target word. The word *badge* has a higher clustering coefficient than the word *log*, because the phonological neighbors of *badge* tend to be neighbors of each other to a greater extent than the phonological neighbors of *log*.

In Experiment 1 of [18] it was found that participants correctly identified words, such as *log*, with lower clustering coefficients more often (72%) than words such as *badge*, with a higher clustering coefficient (58%). Better performance for words with a low clustering coefficient was also obtained in Experiment 2 using the auditory lexical decision task, another conventional and widely used task in psycholinguistics (see also [19,20,21]).

To account for the results in [18], it was suggested that activation would initially spread from the target word to the phonologically related words, and from those words to other words that were phonologically related, and so on. In the case of words with a lower clustering coefficient, the activation would tend to disperse to the rest of the network, allowing the target word to “stand out” from the background of partially activated phonological neighbors, resulting in rapid and accurate retrieval from the lexicon. In the case of words with a higher clustering coefficient, the spreading activation would recirculate among the highly interconnected phonological neighbors, resulting in the target word being “buried” in the background of partially activated phonological neighbors, and therefore slow and less accurate retrieval from the lexicon. In other words, the micro-structure of the phonological lexicon influenced lexical processing.

Although the mechanism proposed in [18] to account for their results was based on computational work performed in other domains of network science, the model they put forward at the time was a verbal model, which have well-known shortcomings compared to computer simulations [30]. Subsequent computer simulations [28,29], however, showed that activation diffusing across 2-hop networks (such as the network displayed in Figure 1) of a different set of stimulus words successfully simulated the behavioral results originally observed in [18], substantiating the original verbal model and demonstrating further that the structure of the phonological lexicon influences lexical processing.

The *initial_lexicon* often used in the TRACE model has 211 words that contain sounds from a restricted set of phonemes, and that have a frequency of occurrence of 20 or more per million in [31]. One could arguably call *initial_lexicon* a “toy” lexicon rather than a lexicon representative of a typical speaker. Note that simulations on jTRACE have used a larger lexicon (*biglex*) of 907 words [32]. Although slightly larger, this lexicon is also not representative of the lexicon of a typical speaker. Rather than using the “toy” lexicon in TRACE or a subnetwork to model the lexicon as in previous simulations examining the influence of clustering coefficient on processing [18], in the present simulations both TRACE and the network model had as their lexicon the 19,340 words that were examined in an initial network analysis of the phonological lexicon [14]. Using the same lexicon not only makes comparison between the two different models equivalent, but the use of a large lexicon also tests if the two approaches can successfully scale up to a lexicon that is more realistic in size and composition to a human lexicon. Granted, estimates of the number of words known by the average person vary widely, but the number of words in the lexicon used in the present simulations is several orders of magnitude larger than the size of the lexicons used in previous computer simulations, arguably making for a more realistic and computationally challenging test of the two types of models.

### 2.1. Materials and Methods

This study was not preregistered. The stimuli used in all of the simulations are listed in Appendix A. The data from the simulations are available upon request from the first author.

jTRACE: The lexicon in the present simulation and the simulations that follow consisted of the 19,340 words in the lexicon examined in [14]. We modified the phonemes and phonetic features in the model to accommodate all of the phonemes that were found in the words in the larger lexicon. Aside from the new lexicon, phonemes, and phonetic features, the default parameters and settings were used for all of the simulations reported here (except Simulation 3b).

Appendix A shows the 76 stimulus words from Experiment 1 of [18] that were presented to jTRACE, which was allowed to process each word for 100 timesteps (N.B., the default setting in jTRACE is 99 timesteps). Although 180 timesteps were used in [18] maximum activation was achieved at approximately 100 timesteps, so we used this smaller number of timesteps in the present simulations to reduce computational burden, thereby accelerating data collection. After 100 timesteps had elapsed we examined the 10 most-activated competitors to obtain the activation level for each of the stimulus words. Although the word with the highest activation value is typically considered to be the word that has been retrieved, we documented the activation level of the stimulus word, even if it was not the most active word in the competitor set. If the stimulus word was not among the 10 most activated competitors, then an activation value of 0 was assigned.

*spreadR*: The lexicon in the present simulation and the simulations that follow consisted of the 19,340 words in the lexicon examined in [14]. This is the same lexicon used in the jTRACE simulations as well. As reported in [33], the network formed from the lexicon contained 19,340 nodes with 31,267 connections placed between nodes if the words differed by the addition, deletion, or substitution of a single phoneme. The giant component of the resulting network contained 6508 (34%) nodes; 10,265 (53%) of the nodes were isolates (i.e., lexical hermits with degree = 0), and the remaining 2567 (13%) of the nodes were found in smaller components (i.e., lexical islands).

The 76 stimulus words from Experiment 1 in [18] were presented to *spreadr* [29] with the following settings for the various parameters in the model. An initial activation value of 20 units was used for each stimulus word in the present simulation. Although *activation* = 100 units in the simulations reported in [28], this value is arbitrary. A smaller value was selected in the present simulations to reduce computational burden, thereby accelerating data collection.

*Decay* (*d*) refers to the proportion of activation lost at each time step. This parameter ranges from 0 to 1, and was set to 0 in the simulations reported here (except Simulation 3b) to be consistent with the parameter settings used in [28].

*Retention* (*r*) refers to the proportion of activation retained in a given node when it diffused activation to other nodes connected to it. This value ranges from 0 to 1, and was set to 0.5 in the simulations reported here. In [28] values ranged from 0.1 to 0.9 in increments of 0.1. Because the various retention values in [28] produced comparable results across retention values, we selected in the present simulations a single, mid-range value (0.5) for the retention parameter in order to reduce the computational burden, thereby accelerating data collection.

The suppress (*s*) parameter in *spreadr* will force nodes with activation values lower than the selected value to activation = 0. It was suggested that when this parameter is used a very small value (e.g., *s* < 0.001) should be used [29]. In the present simulations suppress = 0 in order to be consistent with the parameter settings used in [28].

*Time* (*t*) refers to the number of time steps that activation diffuses or spreads across the network. In [28] *t* = 10; however, in the present simulations *t* = 5. A smaller value was selected in the present case because as shown in Figure 3 of [29], activation values reach asymptote at approximately 5 timesteps, making additional timesteps uninformative. Further, as shown in the hop-plot depicted in Figure 2 in [34] approximately 50% of the network has been reached by traversing on average 5 connections (i.e., hops) in every direction from a given node, suggesting that the network has been sufficiently saturated. We selected in the present simulations a smaller value (*t* = 5) for the time parameter in order to reduce the computational burden, thereby accelerating data collection. At the end of 5 timesteps we documented the activation level of each of the stimulus words.

### 2.2. Results

Given the variety of dependent measures used in the various behavioral experiments that we attempted to simulate in the present study, and the different activation levels in TRACE and the cognitive network model, we attempted in the simulations reported here to replicate only qualitatively the findings from each of the behavioral experiments. For both jTRACE and *spreadr*, larger activation values correspond to better performance in the behavioral tasks (e.g., faster reaction times, more accurate responses, etc.).

In Experiment 1 in [18], words with a lower clustering coefficient were identified more accurately than words with a higher clustering coefficient when presented in noise in a perceptual identification task. In the cognitive network model implemented on *spreadr*, we found that words with a lower clustering coefficient had higher activation levels (*mean* = 1.28 units; *sd* = 0.26) indicating they were identified more accurately than words with a higher clustering coefficient (*mean* = 1.13 units; *sd* = 0.09). An independent samples t-test shows that this difference is statistically significant (*t* (74) = 3.29, *p* = 0.0015).

For jTRACE, activation levels could only be obtained for 2 of the 38 words with higher clustering coefficient (*bath* and *wire*), and no activation levels could be obtained for the 38 words with lower clustering coefficient. For the two words from the higher clustering coefficient condition, both words were the most active items in the candidate set, indicating that they had been correctly retrieved from the lexicon. For the remaining 74 words, the stimulus word was not among the 10 most-active candidates that emerged after 100 timesteps, and was therefore assigned an activation value of zero.

### 2.3. Discussion

The results of the simulation of Experiment 1 in [18] show that the cognitive network model implemented in *spreadr* was able to qualitatively replicate the results obtained in [18]. Specifically, words with lower clustering coefficient were identified more accurately (as indicated by higher activation levels in *spreadr*) than words with higher clustering coefficient. This result not only replicates the behavioral study reported in [18], but also replicates the simulations performed by [28,29] on 2-hop networks using slightly different parameter settings. Given that the cognitive network model successfully replicated the results in [18], one could argue that the structure among the words in the lexicon is important, and may indeed influence processing (lexical retrieval in this case).

Replicating results in a simulation with different parameter settings is one way of qualitatively assessing the global performance of a model [35], making the present simulation with *spreadr* more than a simple replication of previous simulations or behavioral studies. Rather, even though the cognitive network model appears simple on the surface, the present results using different parameter settings in *spreadr* help us better understand the complex behaviors of the model.

One of the major differences between the previous and the present simulation is the significantly larger size of the lexicon/phonological network used in the present simulations. Replicating previous results with a large lexicon suggests that the principles found in the cognitive network approach scale up to a more realistically sized vocabulary.

In contrast, using a more realistically sized lexicon with jTRACE in the present simulation led to performance that was significantly worse than the previous simulation of jTRACE reported in [18] using the toy lexicon often used in TRACE simulations (i.e., *initial_lexicon*). In the previous simulation of jTRACE in [18], the model was able to successfully retrieve from the toy lexicon all 56 stimulus words that varied in clustering coefficient (as measured in the network created from the words in *initial_lexicon*). However, TRACE was not able to differentially retrieve the stimulus words based on their clustering coefficient, thus failing to simulate the behavioral results reported in [18].

In the present simulation, in which jTRACE had a more realistic (i.e., not just high-frequency words) and realistically sized lexicon and was given the task of retrieving the 76 stimulus words used in Experiment 1 in [18], jTRACE was only able to retrieve 2 of the 76 stimulus words, making it difficult to assess whether the model could replicate the results reported in [18]. Is the failure of jTRACE indicative that models of spoken word recognition that “ignore” the structure among words in the lexicon do so at their own peril? Is the performance of TRACE in this case indicative that the representations and processes implemented in the model are problematic, incorrect, or simply do not scale-up to a more realistically sized lexicon?

Perhaps the poor performance of TRACE is just a computational/engineering limitation? Indeed, a new model of spoken word recognition called TISK has been proposed that uses computationally more efficient time invariant string kernels to represent incoming speech input [36]. String kernels are commonly used in machine learning applications to represent sequences of symbols. As noted in [36] (page 4): “To our knowledge, however, there have been no published investigations of string kernels in the domain of spoken word recognition.” Although a model such as TISK may indeed be more computationally efficient than TRACE, it is not clear what such engineering approaches say about human performance or cognitive processing (see also [37,38]).

## 3. Simulation 2: Giant Component/Islands

To examine how the organization of representations in memory at the macro-scale influence cognitive processing we simulated the findings in [23], where words in lexical islands were retrieved more quickly in a naming and a lexical decision task than words located in the giant component. The giant component refers to the largest group of connected nodes in a network. Lexical islands refer to smaller groups of words that are connected to each other, but not to words in the giant component. (“Lexical islands” are referred to simply as “components” in the field of network science.)

### 3.1. Materials and Methods

The same methods and parameter settings used in Simulation 1 for jTRACE and *spreadr* were used in the present simulation. In the present simulation the 96 words used in Experiments 1 and 2 in [23] were presented to jTRACE and *spreadr* (see Appendix A for the words). Forty-eight of the words were found in the giant component, and the remaining words were found in other components/lexical islands in the phonological network.

### 3.2. Results

It was reported in [23] that words located in lexical islands were retrieved more quickly in a naming and a lexical decision task than words located in the giant component of the phonological network. For the cognitive network model implemented in *spreadr*, we found that words located in lexical islands had higher activation levels (*mean* = 5.98 units; *sd* = 2.09) indicating that they were retrieved more quickly than words located in the giant component (*mean* = 3.89 units; *sd* = 1.56). An independent samples t-test shows that this difference is statistically significant (*t* (94) = 5.56, *p* = 0.0001).

For jTRACE, activation levels could only be obtained for 2 of the 48 words located in the lexical islands (*beckon* and *lizard*), and no activation levels could be obtained for the 48 words located in the giant component. For the two words located in the lexical islands, one word was the most active item in the candidate set (indicating that it had been correctly retrieved from the lexicon), and the other word was simply among the 10 most-active candidates, but was not the most active candidate. For the remaining 94 words, the stimulus word was not among the 10 most-active candidates that emerged after 100 timesteps, and was therefore assigned an activation value of zero.

### 3.3. Discussion

The results of the present simulation show that the cognitive network model was able to qualitatively replicate the results obtained in Experiments 1 and 2 in [23]. Specifically, words located in lexical islands were retrieved more quickly in a naming and a lexical decision task (as indicated by higher activation levels in *spreadr*) than words located in the giant component of the phonological network (see also [39]). As in Simulation 1, TRACE did not recognize most of the stimulus words, making it difficult to assess if TRACE can replicate the results of [23].

Given the success of the cognitive network model, the present result may again suggest that the structure of the lexicon has an important influence on processing. Indeed, the structure of the phonological network is responsible for the higher activation levels obtained for the words in the present simulation compared to the activation levels obtained for the words in Simulation 1. Recall that lexical islands are groups of words that are connected to each other, but not connected to the giant component. In the giant component there are many more words for activation to spread to [34], resulting in less activation remaining in the target words in the giant component. In the lexical islands, however, which are smaller than the giant component, the activation will spread among the words in the island, but because there is nowhere else to spread to, activation will remain trapped in the island, resulting in relatively higher activation levels for the words in the present simulation compared to the activation levels obtained in Simulation 1.

## 4. Simulation 3a: Key Players

**Figure 3:**
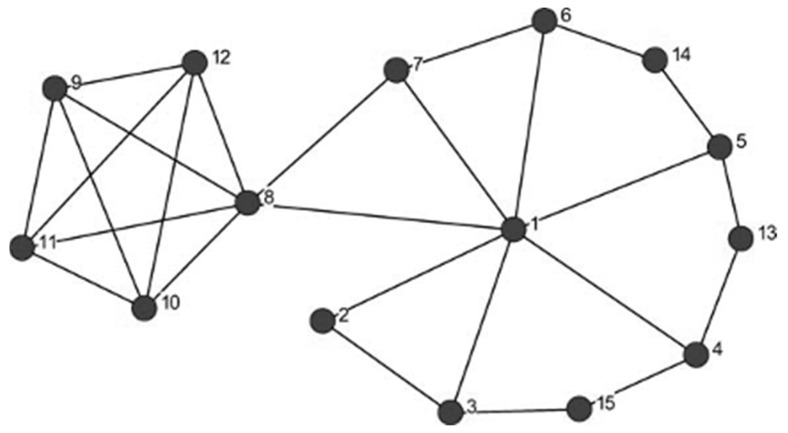
Node 8 is a key player in this network because the removal of that node results in the disconnection of the network (i.e., instead of there being the single component depicted above, two smaller components are formed).

To examine structure at the meso-scale of the phonological network we simulated the results of [24], who examined how a set of words in “key” positions in the network might influence lexical processing. When asked to identify a node in a “key” position in the network in Figure 3, many people select node 1, because it is connected to many other nodes in the network. In network science terms, node 1 has high degree centrality. In contrast, the Keyplayer algorithm developed by [40] would identify node 8 as being in a “key” position in this network, because when node 8 is removed from the network the network becomes disconnected, forming two smaller components.

It was reported in [24] that a set of words in key positions (such as node 8 in Figure 3), whose removal would disconnect the network, tended to be responded to in the lexical decision task used in Experiment 3 more quickly than foil words. Foil words were similar to the set of keywords in word frequency, neighborhood density, word length, and a variety of other lexical characteristics; they just were not located in those key positions in the network.

Because of their strategic position in the network, it was suggested that words in those key positions would be indirectly and partially activated more often than words not in key positions when nearby words were retrieved [24]. Over time that indirect and partial activation from nearby words might, for example, lower the activation threshold or raise the resting activation level of keywords more than foils, making the keywords easier to retrieve than comparable words that were not in those key positions.

### 4.1. Materials and Methods

The same methods and parameter settings used in the previous simulations for jTRACE and *spreadr* were used in the present simulation. In the present simulation, the 50 words used in the three experiments reported in [24] (see Appendix A) were presented to jTRACE and *spreadr*. Twenty-five of the words were in key positions, and the remaining words were referred to as foil words. As reported in [24], the foil words were comparable to the key words in word length, subjective familiarity, word frequency, neighborhood density, neighborhood frequency, phonotactic probability, the duration of the stimulus sound files, clustering coefficient, and closeness centrality. All of the words were found in the giant component of the phonological network.

### 4.2. Results

It was reported in [24] that a set of words in key positions (such as node 8 in Figure 3), whose removal would disconnect the network, tended to be responded to more quickly and accurately than foil words. For the cognitive network model (implemented on *spreadr*) we found that key words had activation levels (*mean* = 2.65 units; *sd* = 0.82) that were statistically indistinguishable from the foil words (*mean* = 2.63 units; *sd* = 1.46; *t* (48) = 0.05, *p* = 0.9544).

For jTRACE, activation levels could only be obtained for 2 of the 25 key words (*amend* and *auricle*), and for 4 of the 25 foil words (*album, aloft, attest*, and *party*). For the two key words both stimulus words were the most active item in the candidate set (indicating that they had been correctly retrieved from the lexicon). For the four foil words, two were the most active item in the candidate set, and two were simply among the 10 most-active candidates (but were not the most active candidate). For the remaining 44 words, the stimulus word was not among the 10 most-active candidates that emerged after 100 timesteps, and was therefore assigned an activation value of zero.

### 4.3. Discussion

As in the previous simulations, TRACE did not recognize most of the stimulus words, making it difficult to assess if TRACE can replicate the results of [24]. We note, however, that TRACE performed better in this simulation than in the other simulations, successfully retrieving six words compared to two words in Simulation 1 and two words in Simulation 2.

Although it was found in [24] that words in key positions were responded to more quickly than foil words, the cognitive network model with the same parameters as used in the previous simulations was not able to simulate that finding. Recall that a verbal model was proposed in [24] that suggested that words in key positions would be indirectly and partially activated more often than words not in key positions when nearby words were retrieved. Over time that indirect and partial activation might, for example, lower the activation threshold or raise the resting activation level of key words, making them easier to retrieve than comparable words that were not in those key positions. Several examples in the literature that demonstrated that partial activation of competitors can affect subsequent processing were discussed in [24], but the observed effects were not computationally modeled.

Our attempt in the present simulation to model the effects observed in [24] overlooked the crucial mechanism of partial and indirect activation modifying subsequent ease of lexical access. Because *spreadr* simply performs a single retrieval event and does not have a mechanism in it to allow for previous retrievals to affect subsequent retrievals, it should not be surprising that the cognitive network model implemented with the current set of parameters in *spreadr* was not able to reproduce the results observed in [24] with human listeners. Further, given all of the lexical variables that were comparable in the key and foil words, it should not be surprising that *spreadr* retrieved both sets of words with comparable (and statistically indistinguishable) ease. In the next simulation, we manipulated one of the other parameters in *spreadr* to try to mimic the changes that occur to keywords over time that were proposed in [24].

## 5. Simulation 3b: Key Players Manipulating Decay in *spreadr*

Recall that it was suggested in [24] that words in key positions in the phonological network would be indirectly and partially activated more often than words not in key positions when nearby words were retrieved. Over time indirect and partial activation from nearby words might, for example, lower the activation threshold or raise the resting activation level of keywords more than foils, making the keywords easier to retrieve than comparable words that were not in those key positions.

Another alternative not discussed in [24] is that previous or partial activation of a node might also influence subsequent activations of that node not by directly “strengthening” the node (i.e., lowering the threshold, or raising the resting activation level), but by “strengthening” the connections to the node. Indeed, such a mechanism is described in Node Structure Theory (NST), a model of language processing proposed in [41]. In NST the connections between nodes become stronger or more efficient with use, enabling the rate and amount of priming transmitted across the connections to increase over time. (Note that “priming” in NST is akin to spreading activation in other types of models.) It is this mechanism that allows NST to account for the well-known effects of the frequency of occurrence of a word in language processing.

To alter the efficiency of the connections in the phonological network, thereby affecting the rate and amount of activation that diffuses through the network, we decided to manipulate the *decay* (*d*) parameter in *spreadr*. The *d* parameter determines the proportion of activation that is lost at each time step. More efficient (or stronger) connections should lose a small amount of activation at each time step, whereas less efficient (or weaker) connections should lose a larger amount of activation at each time step. This parameter ranges from 0 to 1, and was set to 0 in the previous simulations to be consistent with the parameter settings used in [28]. In the present simulation we manipulated d in an attempt to vary the efficiency of the connections for the foil and key words, similar to the mechanism in NST put forward in [41]. Based on the argument in [24] that keywords become “stronger” than foils over time, we set in the present simulation *d* = 0.1 for the keywords and *d* = 0.3 for the foils, but the rest of the parameters remained as they were in the previous simulations.

Given that we changed a parameter in *spreadr*, we decided to try a different parameter setting for TRACE as well. The performance of TRACE across the three previous simulations was best in Simulation 3a, with six words being successfully retrieved from the lexicon. That level of performance will provide us with a reasonable baseline to allow us to determine if different parameters in TRACE would increase or decrease the number of words it successfully retrieved from the lexicon (and perhaps even allow us to evaluate whether TRACE can account for the behavioral finding being simulated). As noted in endnote 2 in [25] (page 30), there is some risk involved in changing parameters in TRACE:

*As Frauenfelder once put it in a conference presentation [42], the large number of parameters in TRACE are in “delicate equilibrium.” Caution must be exercised when changing any parameters, since a small change in one parameter may result in large changes in the model’s behavior, and one cannot be sure that the model will successfully perform simulations conducted with other parameter settings.*

Heeding this warning, we therefore decided to change just one parameter in jTRACE; namely, we turned off lexical feedback. This parameter was also turned off in the simulations reported in [18] to test if a different model of spoken word recognition—Shortlist [8], which eschews feedback—could account for the behavioral results that they found (and which were replicated in Simulation 1 reported here). Like the TRACE simulation reported in [18], the Shortlist simulation successfully retrieved all of the words from the toy lexicon that was used, but did not have differential activation values for the words that varied in clustering coefficient.

We recognize that there is debate about the utility of lexical feedback in TRACE. For example, it was reported in [43] that reducing feedback from 0.030 to 0.025 improved performance with a larger lexicon of 977 words (referred to as *Biglex*), and that turning off lexical feedback sped recognition time for about half of the small set of words (*n* = 21) they examined. In contrast, it was reported in [32] that when a larger set of words (*n* = 900) was examined (without noise), 27% of the words were recognized more quickly without feedback, 57% were recognized more quickly with feedback, and 16% had equivalent retrieval times with and without feedback. It was also observed in [32] that feedback increased accuracy when increasing levels of noise were added to the input. Given that we are not adding noise to the input in the present simulation, and given the partial success of turning off lexical feedback reported in [18], we decided to examine if turning off lexical feedback might improve performance when TRACE has the much larger lexicon being used in the present simulations.

### 5.1. Materials and Methods

The same methods and parameter settings used in the previous simulations for jTRACE and *spreadr* were used in the present simulation, with the exception of *decay* (*d*) being manipulated in *spreadr*, and lexical feedback was now turned off in jTRACE. The 50 words used in the three experiments reported by [24] (see Appendix A) and in Simulation 3a were presented to jTRACE and spreadr in the present simulation. Twenty-five of the words were in key positions (and had the *decay* parameter, *d*, set to 0.1 in *spreadr*), and the remaining words were referred to as foil words (and had the decay parameter, *d*, set to 0.3 in *spreadr*) to mimic the change in processing efficiency that occurs over time proposed by [24].

### 5.2. Results

It was found in [24] that a set of words in key positions (such as node 8 in Figure 3), whose removal would disconnect the network, tended to be responded to more quickly and accurately than foil words. For the cognitive network model implemented in *spreadr*, we found that key words (with the *decay* parameter *d* = 0.1) had higher activation levels (*mean* = 1.57 units; *sd* = 0.49) indicating that they were retrieved more quickly than the foil words (with the *decay* parameter *d* = 0.3; *mean* = 0.44 units; *sd* = 0.25). An independent samples t-test shows that this difference is statistically significant (*t* (48) = 10.29, *p* < 0.0001).

For jTRACE with no lexical feedback, activation levels could be obtained for 3 of the 25 key words (*amend, auricle* (the same words retrieved in Simulation 3a), with the addition of *pallet*), and for 4 of the 25 foil words (*album, aloft, attest*, and *party*; the same words retrieved in Simulation 3a). For both the foil and key words, all of the words were the most active item in the candidate set. Instead of assigning zero activation to the remaining items, in this simulation we simply compared the mean activation values for the three key words (*mean* = 0.6585; *sd* = 0.006) to the mean activation values for the four foil words (*mean* = 0.6700; *sd* = 0.009) that jTRACE successfully retrieved. The difference in activation levels was not statistically significant (*t* (5) = 1.76, *p* = 0.14). Further, the direction of the difference was the opposite of what was predicted based on the behavioral results reported in [24].

### 5.3. Discussion

It was found in [24] that participants responded to words in key positions more quickly than foil words. They accounted for that result by suggesting that words in key positions would be indirectly and partially activated more often than words not in key positions when nearby words were retrieved. Over time that indirect and partial activation might, for example, lower the activation threshold of key words, raise the resting activation level of key words or, as suggested in NST [41], might strengthen or increase the efficiency of the connections between nodes for keywords, making keywords easier to retrieve than words that are not in those key positions.

To mimic the differences in connection efficiency as suggested in NST [41] we manipulated in this simulation the decay (*d*) parameter in *spreadr*. With the manipulation of *d* in the present simulation (compared to Simulation 3a) we now observed that words in key positions with more efficient/stronger connections were responded to more quickly than foil words with less efficient/weaker connections (as indicated by higher activation levels for keywords compared to foils). The result of Simulation 3b qualitatively replicates the behavioral result observed in [24].

We also manipulated a parameter in jTRACE in an attempt to improve the performance of the model. In this case, we turned off lexical feedback, which did improve performance. In the present simulation seven words were retrieved, compared to six words in Simulation 3a. Further, all of the words that were retrieved in the present simulation were actually the most active item in the candidate set (compared to only four of the six words being the most active item in the candidate set in Simulation 3a). Although there is some debate about whether feedback improves performance in TRACE (cf., [32,43]), in the present case turning off lexical feedback did improve the overall performance of the model with a larger set of phonetic features and phonemes, and a much larger lexicon. Although the overall performance of TRACE was improved by the manipulation of this parameter, the difference in the activation values of the words that were retrieved was not statistically different, and trended in the opposite direction to what was predicted based on the behavioral results reported in [24].

## 6. Conclusions

In the present study we simulated in TRACE [7,25] and a phonological network [14] using the R package *spreadr* [29] the results of three psycholinguistic experiments that examined how the structure of a phonological network at the micro-, macro- and meso-scale might influence lexical retrieval. At the micro-scale (Simulation 1), measuring the characteristics of individual nodes, we simulated the results in [18] examining the measure known as the (local) clustering coefficient, which measures the extent to which neighbors of a word are also neighbors of each other. At the macro-scale (Simulation 2), measuring the characteristics of the entire network, we simulated the results of [23] who looked at whether a word being located in the giant component or in a lexical island influenced lexical retrieval. At the meso-scale (Simulations 3a,b), which considers groups or subsets of nodes rather than individual nodes or the whole network, we simulated the results of [24] who looked at how key players in the network might influence lexical processing. Key players refer to a set of nodes whose removal from the network results in maximal disconnection of the network.

In Simulations 1 and 2 the cognitive network model qualitatively replicated the results observed in the psycholinguistic experiments, but TRACE was not able to successfully retrieve a sufficient number of words to assess the ability of this model to simulate the behavioral results. In Simulation 3a the cognitive network model was not able to successfully replicate the results observed in the psycholinguistic experiment. In this simulation, TRACE was able to successfully retrieve a larger number of words compared to the previous simulations, but still not enough words to assess statistically the ability of this model to simulate the behavioral results.

The failure of the cognitive network model in Simulation 3a led us to reconsider the mechanism proposed in a verbal model in [24]: previous activation and retrievals of nearby words can influence subsequent retrievals of the target word. Although verbal models are useful in the initial stages of a theory, many have written about the value of using formal, computational models to more precisely examine the representational and processing aspects of cognition [30]. Therefore, in Simulation 3b we manipulated another parameter in *spreadr*, namely the *decay* (*d*) parameter to mimic the changes that occur over time to the key words. When the keywords and foils had different values for the *d* parameter to model differences in the strength/efficiency of the connections to those words, we now observed a qualitative replication of [24] in the cognitive network model.

Given that we manipulated a parameter in *spreadr* in Simulation 3b, we decided to also manipulate a parameter in jTRACE to see if performance could be improved enough to assess the ability of the model to qualitatively replicate the results of [24]. In Simulation 3b, we turned off feedback from the word level to the phoneme level as had been carried out in [18] and in [43] (cf., [32]). Here we found that overall performance did improve enough to statistically analyze the activation values of the key and foil words. However, the difference in the activation values was not statistically different.

The poor performance of TRACE in the present set of simulations is troubling, especially given that [7] (p. 22) reported that the behavior of TRACE was qualitatively robust over a wide range of parameter values (with minor changes in the magnitude or timing of various effects when using different parameter settings). We grant that the default parameters in TRACE established with the original, very small lexicon and a limited set of phonetic features and phonemes may be optimal only under those conditions. We further grant that in the present situation with different and larger sets of phonetic features and phonemes, and a much larger lexicon, that the default parameters may be suboptimal. However, even shutting off lexical feedback as we did in Simulation 3b did little to change the performance of the model.

Others have discussed the importance of testing model performance across a range of parameter settings [35], and of assessing the scope of a model [44]. Here we simply note that the cognitive network model in Simulation 1 of the present study performed accurately with different parameter settings than used previously [28,29], suggesting that the performance of cognitive network models may not be as sensitive to a unique set of parameter settings as other types of models.

We believe there is much to learn about cognition by using formal, computational models [45] as long as realistic contexts rather than idealized or over-simplified settings are used in those models [46]. Recall that in the TRACE simulation reported in [18] the model successfully retrieved from the toy lexicon all of the stimulus words that varied in clustering coefficient (as determined by measuring the clustering coefficient of the 211 words in the *initial_lexicon*), but no difference as a function of clustering coefficient was observed. In the present simulations where TRACE and *spreadr* were given a more realistically sized lexicon of 19,340 words, TRACE retrieved such a small fraction of the stimulus words that statistical analyses could not be performed in most cases. In contrast, the cognitive network model was able to scale-up from smaller subnetworks (i.e., the 2-hop networks used in [28,29]) to a lexicon that was several orders of magnitude larger, demonstrating the robustness of the cognitive network approach in simplified and in more complex/realistic settings.

In addition to highlighting the importance of using formal, computational models to increase our understanding of cognition, the results of the present study suggest that future models of cognitive processing should consider how representations are organized in memory, and how that structure influences processing. The psycholinguistic experiments simulated in the present study demonstrated that the structure of a phonological network at multiple scales (micro, meso, and macro) influences various language processes. We believe the influence of structure on processing also applies to other areas of Cognitive Psychology [21]. Indeed, experiments from cognitive science, neuroscience, and linguistics demonstrate that humans are able to learn about the meso- and macro-scale of network representations in memory, and that those structures influence processing in a variety of cognitive tasks [47].

The present results suggest that what cognitive networks “do” is capture the regularities and relationships that exist among representations in memory. With the “smarts” of the system captured in the structure of the network (i.e., how the nodes are connected to each other), a much simpler processing algorithm—such as a random walk, the diffusion of activation across the network [27], or a mixture of random and directed walks [48]—may be sufficient to reproduce the behavior exhibited by humans in various tasks [49].

Cognitive networks may not be the only way to model the regularities and relationships that exist among representations in memory, and how that structure influences processing. Indeed, just as there are limits to what a network can model in other domains, there may be some limits to what cognitive networks can model [50]. Similarly, the richness and variety of cognition may be too complex to be captured by simple processes such as a random walk or diffusion of activation across the network.

In addition, the present simulations used a cognitive network that captured a “snap shot” of only the phonological lexicon of the “average” language user at one point in time. Advances in network science and in the application of networks to psychology are rapidly being made to address some of these limitations inherent in the present simulations. Work reported in [51] demonstrated that networks that grow over time can be used to provide insight into how typically developing and “late talking” children learn the meanings of new words. A similar approach has been used in [52] to capture changes/declines in semantic information in older adults. These studies also demonstrate that cognitive networks may hold much promise for increasing our understanding of various speech, language, and hearing disorders as well [39].

Cognitive networks need not be limited—such as the phonological network examined in [14]—to one type of representation or information. Work on multilevel networks, which enable researchers to look at, for example, a network of words with phonological relationships overlayed on a network of words with semantic relationships have increased our understanding of word-learning in children [53], and of acquired language disorders in adults [54].

Further, increasingly sophisticated network analyses are providing tools to track changes in behavior over time in an individual, rather than the average behavior of a group [55]. Such analysis techniques have significant implications for individualized- or personalized-treatment in a number of domains (e.g., psychopathology, speech-language-hearing disorders, etc.).

Although the TRACE I and TRACE II models accounted for a wide range of phenomena in speech perception and spoken word recognition [7], we find it troubling that the most successful model of spoken word recognition did not scale up to a more realistic lexicon. Further, as described above, we believe that the cognitive network approach has much potential to account for an increasing number of phenomena in speech perception and spoken word recognition, as well as other areas of Cognition. Furthermore, the cognitive network approach may not only account for the same phenomena in speech perception and spoken word recognition that the TRACE models can [56], but may also account for phenomena that TRACE and other contemporary models of spoken word recognition cannot account for, such as the influence that the structure of the lexicon at various scales has on processing.

## Data Availability

The data from the simulations are available upon request from the first author.

## Appendix A

**Table A1:** Stimulus words from Chan and Vitevitch (2009) that were used in Simulation 1.

High Clustering Coefficient	Low Clustering Coefficient
bash	beach
bath	bead
bib	beat
bull	bush
bug	boot
dot	dog
dig	dead
dish	deck
dug	debt
feel	fat
full	fell
foul	fate
gang	gas
gain	goat
gum	gull
call	cough
case	couch
lag	lock
leaf	log
leap	lose
lease	ledge
leave	lick
look	lip
lose	live
lull	lime
love	luck
math	miss
mall	merge
meal	mood
mouse	mile
perk	pass
pearl	purse
ring	rhyme
ripe	rise
seal	sauce
size	save
weak	word
wire	wide

**Table A2:** Stimulus words from Siew and Vitevitch (2016) that were used in Simulation 2.

Giant Component	Lexical Islands
brittle	banish
cartridge	beckon
ceiling	central
century	coffin
chapter	concede
colleague	concern
collect	confine
comic	consign
coroner	cunning
cumber	deafen
danger	domain
defend	felon
device	furnish
drench	gallop
dribble	happen
driven	lizard
facet	locus
filing	manage
grunt	margin
hamper	marriage
hardly	memory
knowledge	mission
languor	nervous
limber	nominee
magnet	notice
mention	partition
minute	peasant
mountain	permission
mustard	petition
panther	plaza
parable	portion
parcel	position
receive	radio
remind	regain
remit	remain
repeat	report
reverse	retail
rollick	retain
salvage	revolve
scant	service
scepter	siphon
spiral	soften
squid	solemn
straighten	taken
stutter	treasure
supposed	trophy
temple	village
temporal	warrant

**Table A3:** Stimulus words from Vitevitch and Goldstein (2014) that were used in Simulation 3a,b.

Keywords	Foils
Amend	Album
Auricle	Aloft
Bring	Attest
Colic	Brief
Defy	Cockney
Filing	Downy
Fish	Espy
Inurn	Firm
Leva	Feudal
Ling	Lave
Lion	Lighten
Milling	Manna
Misty	Mystic
Opine	Osprey
Over	Party
Packet	Pasty
Pallet	Pilot
Pocket	Poster
Polite	Rent
Scrawl	Rupee
Spring	Squirt
Tenet	Stilt
Tense	Test
Void	Torrid
Wrist	Vest
